# Designing learning technology collaboratively: Analysis of a chatbot co-design

**DOI:** 10.1007/s10639-022-11162-w

**Published:** 2022-06-24

**Authors:** Eva Durall Gazulla, Ludmila Martins, Maite Fernández-Ferrer

**Affiliations:** 1 INTERACT Research Unit, University of Oulu, Oulu, Finland; 2 Learning, Media & Social Interactions, Universitat de Barcelona, Barcelona, Spain; 3 Learning, Media & Social Interactions, Universitat Oberta de Catalunya, Barcelona, Spain

**Keywords:** Collaborative design, Learning technology, Technology-enhanced learning, Conversational interface, Chatbot, Action research

## Abstract

Collaborative design approaches have been increasingly adopted in the design of learning technologies since they contribute to develop pedagogically inclusive and appropriate learning designs. Despite the positive reception of collaborative design strategies in technology-enhanced learning, little attention has been dedicated to analyzing the challenges faced in design processes using a collaborative approach. In this paper, we disclose the collaborative design of a chatbot for self-regulated learning in higher education using an action research approach. We analyze the design process of EDUguia chatbot, which includes diverse evidence from questionnaires and workshops with students and lecturers, as well as intermediary design objects. Based on the qualitative analysis, we identify several challenges that are transversal to the co-design work, as well as specific to the design phases. We critically reflect on the strategies deployed to overcome these challenges and how they relate to decision-making processes, highlighting the need to make stakeholders’ tacit knowledge explicit, cultivate trust-building and support democratic decision-making in technology design processes. We believe that the recommendations we present in this paper contribute to developing best practices in the collaborative design of chatbots for the self-regulation of learning, as well as learning technology in general.

**Supplementary Information:**

The online version contains supplementary material available at 10.1007/s10639-022-11162-w.

## Introduction

In recent years, scholars have advocated supporting student participation in the design process to develop learning designs that are pedagogically inclusive and appropriate (Villatoro & de Benito, 2021). In Technology-Enhanced Learning (TEL), involving students during the design process has been also considered beneficial for avoiding feelings of alienation, especially when exploring the potential of emerging technologies for teaching and learning such as automatic data collection systems and chatbots (Durall Gazulla et al., 2020; Durall & Kapros, 2020).

The democratization of educational technology design processes has been considered a promising strategy to develop high-quality teaching and learning (Fleischmann, 2015). From a technology design perspective, it has been claimed that collaborative design processes lead to solutions that are more flexible and solid in use, accessible to a greater number of people, and more adaptable to changing situations over time (Gros & Durall, 2020). Designing technology in a collaborative way implies generating collective processes in which all the stakeholders involved have the possibility of influencing and controlling the process and the solutions generated through it (Ehn, 1988). For collaborative design to be successful, it is key to establish horizontal relationships of mutual learning in which stakeholders’ knowledge and contributions are valued (Druin, 2014). For this to happen, trust-building and supporting joint negotiation have been also recognised as important aspects to consider in collaborative design processes (Robinson & Simonsen, 2012; Stelzle et al., 2017).

While scholars have strived to open collaborative design processes, examining aspects such as decision-making and power tensions, as well as how to reach agreements and support empowerment (Bønnelycke et al., 2018; Bratteteig & Wagner, 2012; Ertner et al., 2010; Spiel et al., 2020), in learning technology design, little attention has been paid to how design decisions are taken in collaborative processes. We believe there is a need for evidence-informed debate on the challenges that appear at different stages of collaborative design processes and how these have an impact on major and minor decisions leading to the definition of design requirements. This is important because technologies are not value-neutral (Bucher, 2018; Feenberg, 2017) and thus design decisions have an impact on what aspects are prioritized in the final design.

This paper analyzes the collaborative design of a chatbot to be used in formal education contexts. Chatbots, also referred to as conversational interfaces, are programs designed to interact with users in a human-like way, answering questions and performing tasks in a specific area (Griol et al., 2017; Winkler & Söllner, 2018). In the educational field, chatbots are increasingly adopted since they support immediate and personalized feedback (Kumar, 2021; Okonkwo & Ade-Ibijola, 2021; Winkler & Söllner, 2018). To date, most common uses of chatbots in education contexts have focused on supporting teaching and learning, administrative tasks, student assessment, and research and development (Okonkwo & Ade-Ibijola, 2021). Despite the reported benefits of using chatbots in education contexts, several challenges have been noticed. In particular, there have been calls for involving education stakeholders in chatbot design processes, avoiding top-down approaches (Durall Gazulla et al., 2020; Durall & Kapros, 2020; Fernández-Ferrer et al., 2021). To the best of our knowledge, there has been no debate around the diverse challenges that might hamper the collaborative design of learning technologies such as chatbots.

In this paper, we present an action research study on the collaborative design of a conversational interface (EDUguia chatbot) for supporting self-regulated learning (SRL) in higher education. We analyze the process, outlining different types of challenges for collaboration that appear at various stages of the design process, as well as some strategies to overcome them. We continue by disclosing how this has had an impact on decision-making, the definition of design requirements and the shaping of the chatbot design. Finally, we discuss our findings, proposing recommendations for supporting collaborative design processes in TEL.

## Background

### Collaborative design of learning technology

In recent decades, a growing number of authors have advocated the use of participatory techniques in learning design (Bonsignore et al., 2013; DiSalvo et al., 2017). Some of the reasons that explain the adoption of this approach in the educational field respond to the predominance of student-centered approaches, as well as the recognition that students are experts of their own learning (Iversen et al., 2017). Influenced by these ideas, TEL scholars have understood collaborative design as part of innovation processes around learning environments and tools (Gros & López, 2016; Treasure-Jones & Joynes, 2018).

From a learning point of view, collaborative design has been linked to constructivist approaches. Both approaches pay attention to the process and the mutual learning of those who participate. From these perspectives, the learning experience is a process that is built over time and in which interaction and dialogue play a fundamental role (Gros & Durall, 2020).

From a technology design perspective, scholars have claimed that participatory and co-design methods are inclusive approaches (Treasure-Jones & Joynes, 2018) that contribute to ensuring that students’ needs are truly taken into consideration. As part of the implications for practice stemming from this work, authors have advocated that “co-design should ideally form part of an iterative, agile development approach” (Treasure-Jones & Joynes, 2018, p.283). Despite the increasing adoption of collaborative design approaches, practitioners have noted some challenges for implementation. Amongst the most prominent ones figure the difficulty for supporting trust-building and balancing tensions (Andersen & Mosleh, 2021; Clarke et al., 2021; Sanders, 2006).

To date, collaborative design has been used to involve teachers and students in technology design (Prieto-Alvarez et al., 2020; Cober et al., 2015; Bovill et al., 2011). The collaborative design of tools for learning has been oriented towards supporting key learning skills such as collaboration (Leinonen & Durall, 2014; Spikol et al., 2009; Tissenbaum & Slotta, 2019), assessment (Harrer & Lingnau, 2018; Penuel et al., 2007), reflection (Durall et al., 2017; Leinonen et al., 2016), self-regulation (Treasure-Jones et al., 2018; Villatoro & de Benito, 2021), as well as self-directed learning (Laanpere et al., 2014).

The existing literature on participatory and co-design approaches in technology design has examined the role and agency of those taking part in collaborative design processes and several tools for analysing participation and involvement in decision-making have been produced (Bratteteig & Wagner, 2016; Koutamanis et al., 2017; Malinverni et al., 2016). However, to the best of our knowledge, none of these studies have focused on the challenges faced in the collaborative design processes in higher education and how these challenges affect decision-making processes. We consider this as important area for further explore to ensure that design of TEL is rooted in democratic tradition.

### Supporting self-regulation through chatbots

From a pedagogical perspective, chatbots can scaffold students’ learning, helping them to address different topics and triggering reflection from the initial questions made by the program. However, chatbots targeting the self-regulation of learning and metacognitive strategies are scarce, which means that there is still potential for the use of chatbots to be explored (Durall & Kapros, 2020; Calle et al., 2020; Scheu & Benke, 2022).

The Council recommendation on key competences for lifelong learning (2018) highlights that learning to learn is one of the key competences to support the training, learning and participation in society in a lifelong perspective. According to the proposal, “Personal, social and learning to learn competence is the ability to reflect upon oneself, effectively manage time and information, work with others in a constructive way, remain resilient and manage one’s own learning and career” (Council of the European Union, 2018, p.10). Deeply connected to the learning to learn competence is the idea that, to be successful, learners should develop a sense of ownership over their learning process (Durall Gazulla et al., 2020). This requires students to be able to self-regulate their learning.

Self-regulation can be defined as self-generated thoughts, feelings, and goal-oriented behaviors, and it is understood as a cyclical process composed of three phases: forethought, performance, and self-reflection (Zimmerman, 2001). Self-regulation of learning is fundamental for the development of lifelong learning skills, and this is one of the main reasons why education should contribute to their development (Zimmerman, 2002). Researchers have pointed out the potential of chatbot technologies for supporting students to self-regulate their learning activity (Fernández-Ferrer et al., 2021; Scheu & Benke, 2022). Recent studies have shown that the use of conversational agents could be useful to support students’ self-regulated learning in online environments (Scheu & Benke, 2022; Song & Kim, 2021).

The work that we present here is framed in a research and innovation project (e-FeedSkill). This research and innovation project builds on self-regulated learning theories, concretely on Zimmerman’s model of SRL (2001), to design a chatbot to be used in higher education environments. As chatbots are still an emerging technology in education contexts, we have adopted a collaborative design approach to help students take ownership of the tool, ensuring that the design aligns with their needs and technology practices. The study reported in this paper aims to contribute to learning technology design practice by following an action research approach that addresses the following questions in the context of a chatbot design:

1. What type of challenges hinder collaborative approaches to the design of learning technologies like chatbots?
2. How to overcome the challenges faced in the collaborative design of a chatbot for supporting SRL?

In order to answer these research questions, in this paper we disclose the collaborative design of a chatbot for SRL, and we critically reflect on the challenges and the strategies we adopted during the design process. In line with research approaches acknowledging the socially situated nature of research (Zhou & Hall, 2018) in this text we use the first person to highlight the authors’ involvement in the study as design practitioners and researchers. We believe that the insights we present in this paper contribute to advance knowledge and support the development of best practices for supporting collaboration and democratic decision-making in the design of chatbots for the self-regulation of learning.

## Methodology

In human–computer interaction, action research has been considered a valuable approach to identifying issues of practice, providing insight on the needs and challenges faced by practitioners (Ogunyemi et al., 2019). Design practice has been connected to action research due to the strong resemblance between both processes. As several scholars have noted, design practice and action research are both oriented towards change and transformation to improve a particular situation through the interplay of action and research moments in successive cycles (Blackler et al., 2018; Swann, 2002). In addition, both action and design research acknowledge the complex interrelations between humans and their context and thus embrace uncertainty and messiness as part of the process (Blackler et al., 2018). In this article, we inquire on the challenges faced by learning technology designers to implement democratic processes in their practice. We advance research on this issue by conducting a qualitative analysis of the collaborative design process of a chatbot for supporting the self-regulation of learning.

**Fig. 1:**
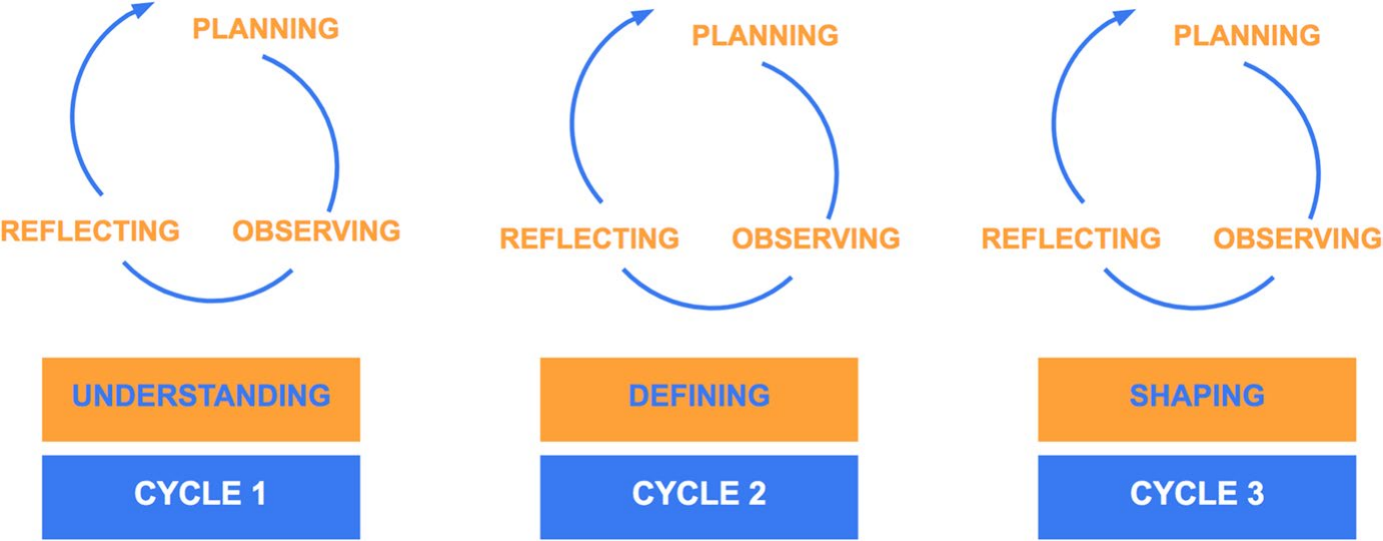
Action research cycles

We reflect on our practice (Schön, 1983) with the aim of better supporting collaboration and democratic decision-making in learning technology design. For this purpose, we have systematically engaged in action research cycles of planning, observing, and reflecting (Kemmis et al., 2014) on each of the design phases of the EDUguia chatbot, which we have defined as *Understanding*, *Defining* and *Shaping* in Fig. 1.

**Fig. 2:**
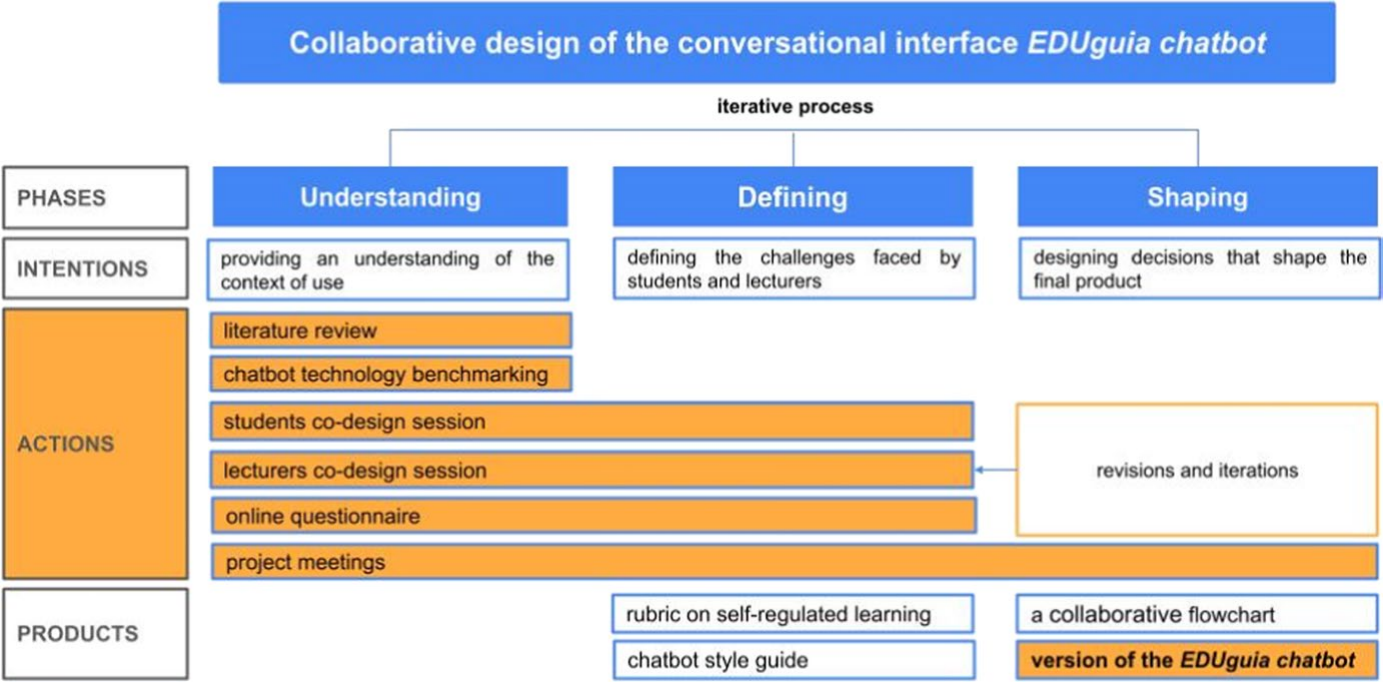
Summary of actions and products

As is common in design processes, design phases are characterized for moving from a fuzzy phase (here referred as *Understanding*) to progressively narrowing the design space (*Defining*) until arriving at a crisp phase (*Shaping*) in an iterative fashion (Design Council, 2007; Tschimmel, 2012; Wolniak, 2017), as it is presented in Fig. 2. The initial phase (*Understanding*) of the EDUguia chatbot design comprised actions intended at providing a general comprehension of the context of use, identifying the stakeholders’ needs and wishes regarding the conversational interface. Some of the actions conducted by researchers and designers during this phase consisted in a literature review on self-regulation and a benchmarking of chatbot technologies, which were further discussed in the project meetings. In these sessions, 43 articles on SRL and 24 on chatbot uses in education published during the five years were selected for close examination. The benchmarking of chatbot technologies led to the selection of five different approaches which were considered for the chatbot design.

During this phase, the expected users, and stakeholders of the EDUguia chatbot were also involved through co-design workshops and questionnaires. In the context of the project e-FeedSkill, these stakeholders were in the first place, the students, and to a lesser extent, the lecturers, since the conversational interface would be piloted during their courses, as an aid for the students’ self-regulation of their academic activity. The insights stemming from *Understanding* were used as the starting point of the *Defining* phase, which focused on the identification of the obstacles faced by students and lecturers for using the chatbot in a learning environment. Parallel to the identification of obstacles, during this phase it was also important to generate and assess potential solutions to overcome the obstacles. The co-design sessions conducted with students and lecturers, as well as the questionnaires provided information that helped to define the design of the conversational interface and identify a list of design requirements. As indicated in Fig. 2, during this phase, intermediate design objects such as a rubric on self-regulated learning and a chatbot style guide were also produced.

The *Shaping* phase can be characterized as a crisp moment, in which the information collected during *Understanding* and *Defining* needs to be translated into design decisions that shape the final product, which in this case was the EDUguia chatbot. Building on the intermediate design objects, a collaborative flowchart to work on the chatbot script was produced. While there is a strong interconnection between the phases, the process of shaping cannot be understood as linear. Before a design decision could be formalized, several adjustments and iterations were conducted. During this phase, a first functional version of the EDUguia chatbot was launched and iterated based on the feedback received through an online questionnaire.

### Participants and context

The study presented in this paper is part of a research and innovation project focusing on the analysis of the effects of feedback supported by digital monitoring technologies on transversal skills (e-FeedSkill). The e-FeedSkill project studies how feedback strategies focused on self-regulation can support learners develop soft skills such as learn to learn and autonomous learning. In the project, the role of technology as a tool for monitoring and supporting learners' activity is also examined through the development and piloting of a chatbot in various university courses.

The chatbot follows a collaborative design approach in which the following stakeholders have been involved:

- Students: 12 undergraduate students from a Spanish university. The selection criteria were based on the studies (at least one student from six different bachelor degrees: Biotechnology, Management and Public Administration, Primary Education, Computer Engineering, Pharmacy and History), course level (with representation of different educational levels), academic record (intermediate performance) and gender.
- Lecturers: Seven lecturers from the six different bachelor’s degrees in which the chatbot would be piloted (Biotechnology, Management and Public Administration, Primary Education, Computer Engineering, Pharmacy and History). In the selection criteria, aspects such as course level, years of teaching experience and gender were considered.
- Researchers: 22 researchers and lecturers from two Spanish universities and with diverse backgrounds (education, biological sciences, IT, administration, social sciences).

### Data collection and analysis

In this study, we have collected diverse data from students and lecturers through questionnaires and online workshops (Table 1).

**Table 1:** Research instruments

Instrument	Participants involved	Data format
Pre-workshop questionnaire	students, lecturers	text
Online workshops	students, lecturers	audio, text inputs
Post-workshop questionnaire	students, lecturers	text

Since the study is framed as action research, we have extended our analysis by examining project communications such as internal team notes, meetings’ minutes, and intermediate design materials such as chatbot drafts, design requirements, the chatbot style guide and the change control document of the chatbot script (Table 2).

**Table 2:** Chatbot intermediate design objects

Intermediate design objects	Participants involved	Data format
Chatbot drafts based on a rubric on Self-Regulated Learning (SRL)	students, lecturers, researchers, designers	text
Chatbot design requirements	students, lecturers, designers, developers	text
Chatbot style guide	designers	text
Chatbot flowchart	designers, developers	visual
Chatbot script change log	designers	text

**Fig. 3:**
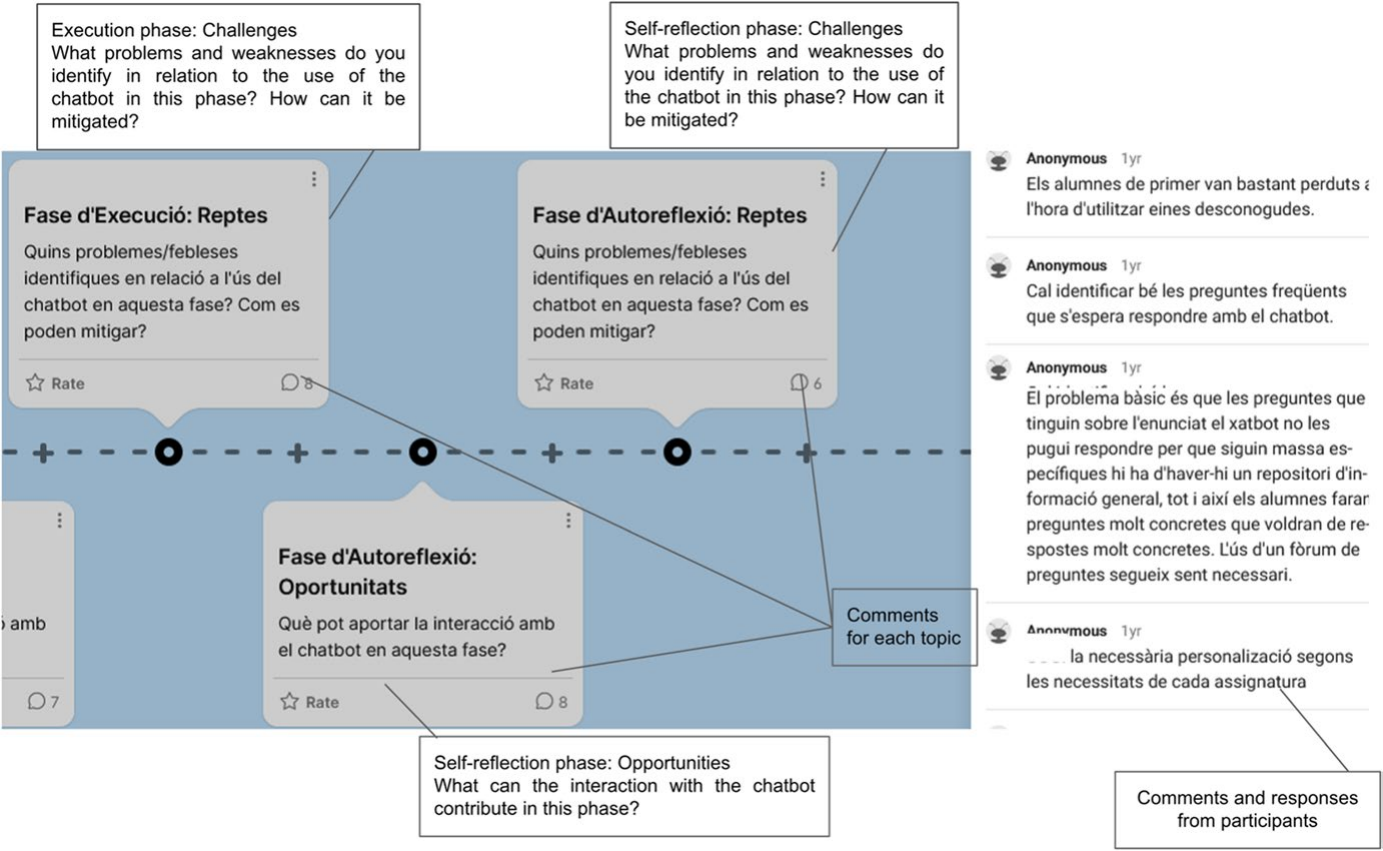
Lecturers’ contributions during the co-design workshop

The project followed a collaborative design approach and thus, it was considered important to capture the ideas, needs and interests of the chatbot’s expected beneficiaries. To this end, two online workshops^1^ were organized with students (n = 12) and lecturers (n = 7) from the studies in which the chatbot was intended to be implemented. Due to Covid-19 restrictions for holding face-to-face sessions, the workshops were run online using visual tools for supporting synchronous participation such as the padlet. At the workshops, students and lecturers were invited to contribute to the padlet boards, by writing their opinions, voting, and expressing their preferences towards the chatbot draft script and contributing to the definition of chatbot opportunities and challenges at different stages of the learning process as shown in Fig. 3.

In the case of the students, the tasks focused on providing insight on their technology preferences, study habits, as well as their wishes, expectations, and concerns regarding the introduction of chatbot tools in their academic activity (Table 3). In the case of lecturers, the workshop tasks oriented at defining the timeline of student-chatbot interactions in the courses in which the chatbot would be implemented and identifying the aspects requiring specific adaptation depending on the knowledge area (Table 3). In both workshops, it was also important to manage expectations regarding the chatbot functionalities.

**Table 3:** Co-design workshops tasks and outputs

Co-design workshop participants	Tasks	Outputs
Students	-Icebreaker on technology preferences -Definition of challenges for developing academic assignments -Discussion on risks and challenges of using chatbots in education -Definition of preferences on the EDUguia chatbot conversation style	-Improved understanding of students’ habits, challenges in their academic activity, and when using technology for learning -Identification of students’ expectations and concerns regarding chatbot technology in academic environments -Selection of the conversation style for the EDUguia chatbot
Lecturers	-Definition of the timeline of student-chatbot interactions during the course -Review of a fragment of the chatbot script	-Identification of challenges and opportunities linked to each of the assignment development phases -Feedback on the chatbot script and generation of self-regulation strategies to be included in the chatbot

For the purposes of this study, we conducted a thematic analysis of the chatbot collaborative design process. Thematic analysis has been described as “a method for identifying, analyzing and reporting patterns within data'' (Braun & Clarke, 2006, p.79). In this study, three researchers have been involved in the phases identified by Braun and Clarke (2006) which include familiarizing with the data, generating initial codes, searching for themes, reviewing themes, defining, and naming themes, and producing a report based on the results. In the following section, we present the findings of the analyses.

## Findings

In this section we identify several challenges arising throughout the collaborative design process of a learning technology, focusing on each of the phases (*Understanding*, *Defining*, and *Shaping*), as well as strategies to support the stakeholders’ understanding and decision-making (Table 4). The evidence presented stems from the EDUguia chatbot collaborative design process, which includes the co-design workshops organized with students and lecturers, the project meetings’ minutes, and intermediate design objects, as well as our reflections as designers and researchers involved in the project.

**Table 4:** Summary of challenges and strategies for decision-making

Type	Challenge	Strategy
Transversal collaboration	-Addressing diverse needs, while ensuring the relevance of the solutions envisioned	-Get information to assess how critical a particular need is and support negotiation between diverse stakeholders
Transversal collaboration	-Supporting stakeholders’ continuous involvement	-Identify key issues that require stakeholders’ input and moments for collaboration -Schedule moment for short feedback (micro-feedback) from stakeholders
Supporting understanding	-Unveiling tacit assumptions	-Ask questions and request clarifications -Build shared vocabularies with stakeholders
Supporting understanding	-Interpreting meanings	-Analyses of data collection through debriefing sessions
Supporting understanding	-Ensuring a certain level of technological literacy among stakeholders	-Provide visual examples
Defining requirements	-Making diverse stakeholders’ needs explicit	-Highlight opposing needs and dilemmas among stakeholders
Defining requirements	-Supporting discussion and finding solutions	-Encourage stakeholders to face conflicts and discuss alternatives
Defining requirements	-Moving from the abstract to the concrete	-Use prototyping to help participants visualize options and show them how different approaches might look -Encourage sharing of best practices among stakeholders to generate solutions that build on existing practices and expertise
Shaping the tool	-Translating research into practice	-Develop intermediary objects to guide the design
Shaping the tool	-Reaching agreements	-Propose concrete solutions and request stakeholders’ feedback through voting and marking elements they like and dislike

### Challenges and strategies for transversal collaboration

The challenges for collaboration vary across the design phases since they are closely related to the objectives of the phase and the tasks at hand. During the design process of EDUguia, and in addition to the phase-related challenges, we also noticed transversal challenges. By using the term transversal, we refer to persistent difficulties and obstacles for collaboration that while manifesting in diverse ways, responded to the same challenge. In this case, we want to highlight two transversal design challenges: the first one being the challenge for addressing diverse needs, while ensuring the relevance of the solutions envisioned, and the second one referring to the challenges for the stakeholders’ continuous involvement.

While we acknowledge the importance of listening to diverse stakeholders’ needs during the design process, sometimes it is not possible to address all of them in the design solution. The challenges for prioritizing among diverse needs relate to various aspects such as assessing the impact that addressing a particular need might have for the rest of the stakeholders. For instance, during the chatbot co-design workshops, some participants expressed very concrete wishes such as using the chatbot to remind them of general information about the course. This expectation conflicted with other approaches based on using the chatbot for skills’ development. Collaboratively assessing to what extent this was a widely shared expectation, as well as the relevance of this approach for other stakeholders was key. In our role as designers, we strived to get information and support negotiation between diverse stakeholders’ needs throughout the design process.

Another challenge for sustaining collaboration throughout the design process related to the stakeholders’ availability to participate in the workshops and provide feedback. This was particularly acute in the case of students since the collaborative activities needed to be scheduled during the academic term without overlapping with their academic activities. Although this challenge refers to logistics, it was a significant obstacle for the stakeholders' participation throughout the project. We worked around this limitation by identifying the key issues that required stakeholders’ input and arranging opportunities for obtaining short feedback.

### Challenges and strategies for supporting understanding

During the early stages of the EDUguia design (*Understanding*), we noticed several challenges for supporting the stakeholders’ mutual understanding and therefore, collaboration. Such challenges related to unveiling tacit assumptions, interpreting meanings, and ensuring a certain technological literacy among stakeholders.

Tacit assumptions refer to implied meanings that are taken for granted and thus, they are not openly mentioned during conversations. While the reasons why these ideas remain unspoken are diverse, making the stakeholders’ tacit assumptions explicit in collaborative design is key to ensure that the chatbot design supports the goals and needs of those who are expected to use the tool.

During the co-design workshops of the EDUguia chatbot, we noticed that some of these assumptions related to fears about technology. For instance, as one of the students involved in the sessions expressed:

“I think it's a double-edged sword depending on how you approach it [the chatbot]. Because if you do it as participant 1 has said, it is fine, but as participant 2 has said, it can remove some of the necessary skills from today's society as it is having a research aptitude. Or we may encourage more shyness or introspection. That is, we may be fostering a very unsocial character, and we may be moving away from people with very negative implications.”

In the online co-design sessions, we were able to create a safe space for students and teachers’ open expression towards technology. The creation of such a space enabled us to ask questions and request clarifications whenever it was needed. As a result of this, we were able to spot the need to develop shared vocabularies with stakeholders. However, these conversations only appeared by the end of the discussion sessions since it took time to understand what the technology could do and how one felt about its potential uses. It is also worth mentioning that some of these fears were closely related to discourses popularized through media (usually movies) about technology.

As designers and researchers, we were in constant need of interpreting the stakeholders’ inputs. The interpretation work happened on-the-go during the synchronous sessions with stakeholders, but also through dedicated moments for analysis such as debriefing sessions with the research and design team. In a way, we might say that during the first phase of the design process, reading between the lines was a key skill for us. Thanks to this interpretative work we were able to gain deeper insights on the students’ strategies for finding support in academic tasks and detecting “patterns” such as the preference for immediate and informal feedback usually through peers, instead of asking the teacher.

Successful communication in collaborative design is also mediated by the stakeholders’ background and their technological knowledge. In this regard, it is important to be aware that previous experiences with a particular technology have a strong impact on potential users’ expectations. During the co-design sessions, it was noticeable that many stakeholders viewed chatbots as personal assistants. For instance, as one of the students shared:

“It [the chatbot] would be especially useful to be more practical and to shorten the procedure when it comes to knowing a formal or normative information, or a recurring doubt. For example, on campus, you could tell us what activities we have to deliver.”

From this perspective, the chatbot benefits consist in supporting efficient access to information, rather than skills’ development. While the assistant metaphor was present among students and lecturers, the last ones pondered and discussed various chatbot approaches with deeper elaboration than the students. Lecturers’ broader imaginaries might be related to their awareness of different chatbot technologies in learning environments, as these had been introduced and discussed in previous project meetings. In this regard, we considered that the uneven awareness of the chatbot possibilities was a challenge for the collaborative design process. During the design of the EDUguia chatbot, we tried to overcome this limitation by using analogies and providing visual examples to stakeholders.

### Challenges and strategies for defining requirements

Before the design requirements are identified there is a progressive process of narrowing down options and identifying alternatives. Even if subtle, the action of defining involves decision-making. During the definition of the EDUguia chatbot design requirements we observed the following challenges for collaboration: making evident for each of the stakeholder groups the needs of other interested stakeholders, supporting stakeholders to discuss alternatives and helping them move from the abstract to the concrete.

In the EDUguia design process, we decided to arrange separate co-design workshops with students and lecturers to ensure students were able to freely express their views without feeling judged by their teachers. Although this approach had benefits such as the obtention of rich feedback about students’ views enabling a more empathic understanding by the design researchers, it also posed some challenges. One of these challenges dealt with the reconciling of opposing needs between stakeholders. For instance, students at the workshop expressed their concerns regarding the constant monitoring of their activity in social and education contexts:

“In the society we live in, we are all under control (...) and that made us a little nervous and uncomfortable. Then we talked about control through the campus and that they can see how many hours you have been there, what documents you work or not, and this was also a bit like constantly monitoring us.”

As the students noted, such a level of monitoring had increased during the covid-19 pandemic during which higher education was moved online. When discussing about using the chatbot in academic activities, the students also showed reluctance to automatic personal data collection and raised questions regarding who would have access to their data. The students’ need for privacy and not being under constant monitoring clashed with researchers’ need to collect data to assess the tool, as well as lecturers’ usage of the chatbot to follow-up students’ individual activity. Making evident for all stakeholders the diversity of needs was challenging due to their limited availability and because these needs opposed each other. From a design perspective, an important challenge consisted in defining whose needs should prevail and finding middle grounds that could accommodate different positions.

At several moments of the collaborative design process, we encouraged stakeholders to face conflicts and discuss alternatives by pointing at opposing needs and dilemmas. While this strategy helped to channel the conversation to address hot topics, moving away from dualistic thinking was hard. For instance, a recurring dilemma throughout the design process was whether the chatbot should include information specific to a particular course or whether it should focus on transversal aspects such as learning to learn skills. As one of the lecturers involved in the co-design workshops expressed:

“I think the content of each subject is very specific and it is difficult to make a chatbot that can cover all this content. I think it should be more about learning. About, for example, how to make a plan, how to make a reflection.”

Moving from the abstract to the concrete is also part of defining. In the co-design sessions, we used prototyping to help participants visualize options and show them how different approaches to the chatbot might look like. As one of the students acknowledged:

“I knew what a chatbot was, but I couldn't imagine exactly what it would be like. And now, looking at it the way it is: asking a question and giving you some options seems pretty good to me. I liked the options and found them to be quite useful.”

In the co-design sessions with lecturers, participants were encouraged to share best practices regarding how to integrate the chatbot in their courses through for instance scheduling dedicated moments to introduce the chatbot and discuss students’ experiences on self-regulation. Building on lecturers’ existing practices and expertise was a valuable strategy to move from the abstract to the concrete, enabling lecturers develop ownership of the chatbot and envision how this new tool would integrate in their teaching.

The analysis of the discussions with students and lecturers taking place during the co-design workshops, as well as of other stakeholders’ wishes and needs informed the definition of the key requirements for the chatbot design. Specifically, the chatbot design requirements consisted in: use across disciplines, theory-based, immediate feedback, easy to understand, brief and smooth interactions, adaptation to existing technology habits, accessible and inclusive, limited personal data collection, data transparency (Table 5).

**Table 5:** List of key requirements for the EDUguia chatbot design

Requirement	Stakeholders making the request	Description
Use across disciplines	Students, lecturers, researchers	Focus on skills development, emphasizing in learning to learn skills, since they were considered relevant and transversal to all fields of knowledge
Theory-based	Lecturers, researchers	Creation of a collection of micro-contents on self-regulated learning, tailoring them to the specificities of the chatbot interaction style
Immediate feedback	Students, lecturers	Organization of the content based on the phase of the self-regulation cycle in which students might be when accessing the tool
Easy to understand	Students	Use of short sentences, plain language, and infographics in the chatbot script
Brief and smooth interactions	Students	Election of a hierarchical tree structure, mostly based on predefined texts. To support self-expression, open text answers were also enabled at certain points
Adaptation to existing technology habits	Students	Based on students’ technology preferences, it was considered they would access the chatbot via a laptop. Thus, the texts and visuals were designed accordingly
Accessible and inclusive	Lecturers, researchers	Accessibility technology standards were followed, ensuring the chatbot could be used by students with diverse needs. Special attention was paid to ensure inclusive language
Limited personal data collection	Students	It was agreed with the research team to reduce data collection to the minimum necessary to assess the tool use during the courses in which it would be tested
Data transparency	Researchers	In addition to the research information provided to guarantee students’ informed consent, an additional message was added at the beginning of the chatbot script, specifying the type of data collected and how it would be used

### Challenges and strategies for shaping the tool

In the EDUguia design process, shaping was grounded on a solid understanding of the socio-cultural context and informed by a careful definition of the stakeholders’ diverse needs and wishes. The key challenges that arose during this phase are related to translating research into practice and reaching agreements.

The EDUguia chatbot was meant to be integrated in a learning environment and thus, it was informed by pedagogical theory. The collaborative design process involved translating theory on self-regulated learning into a tool that connected to the stakeholders’ practices. The development of intermediary documents to guide the design such as rubrics and a chatbot style guide supported communication with researchers while helping to shape the chatbot design. For instance, the chatbot style guide included specific guidelines such as the suggested length of the messages, tone and type of questions, use of emojis and links for writing gender-neutral texts. The guide also included aspects to be avoided such as complex phrasing, academic jargon or questions that might be interpreted by the students for control or evaluation purposes.

The intermediary objects were the basis for the design of the EDUguia chatbot functional prototypes, which were shared for feedback and revised accordingly. The decisions made throughout the collaborative design process have led to the EDUguia chatbot, which is a conversational interface for supporting the self-regulation of learning in student-led academic tasks. The tool targets higher education students from all fields of knowledge and is meant as an aid for developing learning to learn skills.

**Fig. 4:**
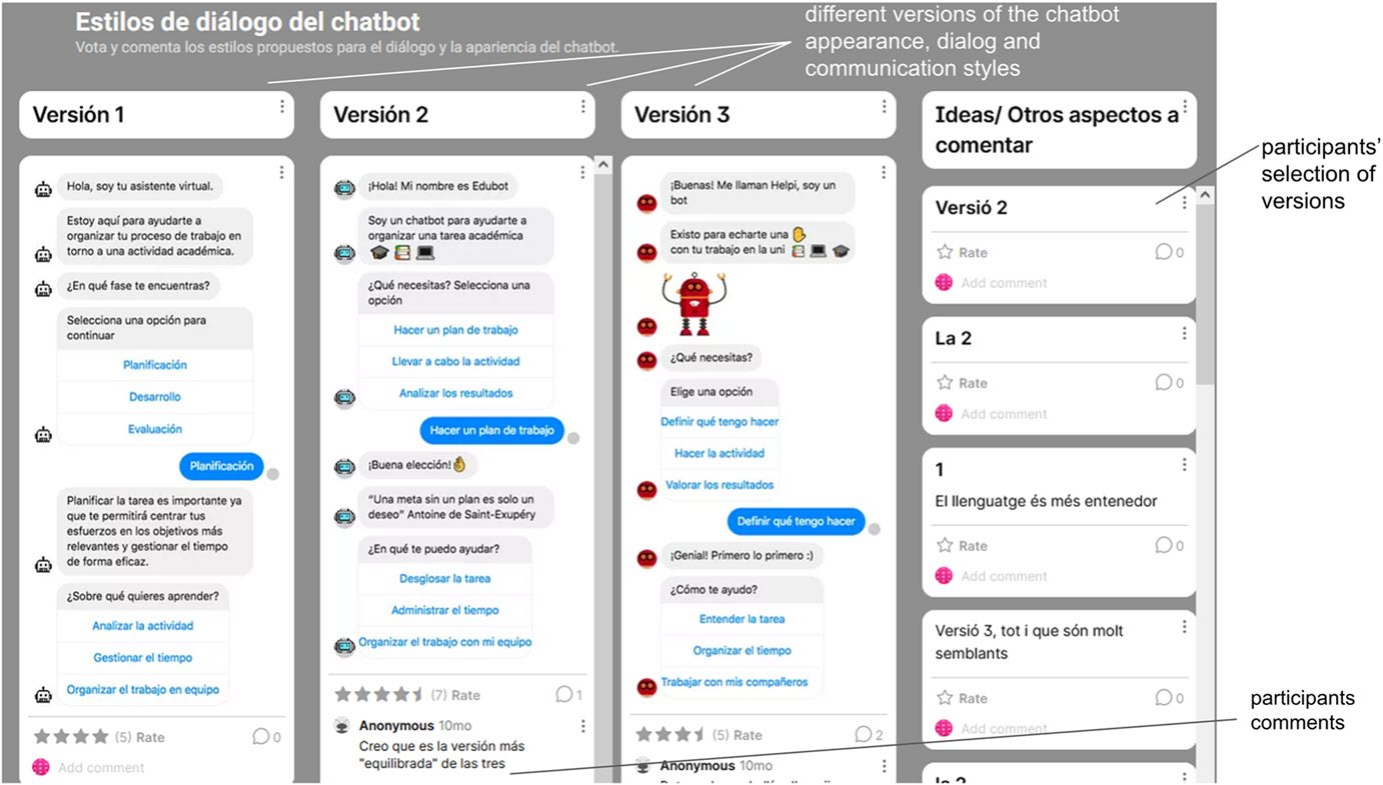
Padlet of the EDUguia chatbot conversation styles

As designers, ensuring progress in the shaping of the EDUguia chatbot was part of our duties. To accomplish this task, we looked for ways to foster decision-making and support consensus. Thus, at given moments we aimed to close open debates by proposing a specific solution and requesting feedback from stakeholders. In some cases, we invited participants to vote between different options as shown in Fig. 4, asking them to indicate their preferences towards the chatbot draft script.

Based on the data collected during the co-design workshops, it was decided that the chatbot would accompany all the phases of the self-regulated learning cycle: the establishment of goals, the understanding of the criteria, the reflection on how the task is carried out, the redirection of strategies, the search for efficiency in the development of the activity and the assessment of the result achieved. At the beginning of each EDUguia session, students would indicate the phase they considered they were at regarding an academic task. Depending on the phase, students could access resources for supporting goal setting, reflecting on the task progress, and assessing the results achieved. For instance, when planning an activity, students could use the chatbot to find information about how to define objectives and set learning goals, organize their work, cope with obstacles, manage time and avoid distractions. During the task execution, the chatbot would include resources to help students monitor their progress, managing their time and emotions, as well as gaining awareness about key skills for learning. During this phase, special attention would be also paid to stress management and sustaining interest and motivation. Once students finalized the academic task, the EDUguia would encourage them to self-assess their performance, reflect, and learn from their mistakes, as well as carefully consider their emotions, feelings and think about how these reflections might impact further learning endeavors. These resources were expected to have effects on the cognitive components (memory, attention, problem solving) and, especially, the metacognitive (understanding of the learning process itself and thought processes) and affective (emotions, feelings, etc.) dimensions of learning.

**Fig. 5:**
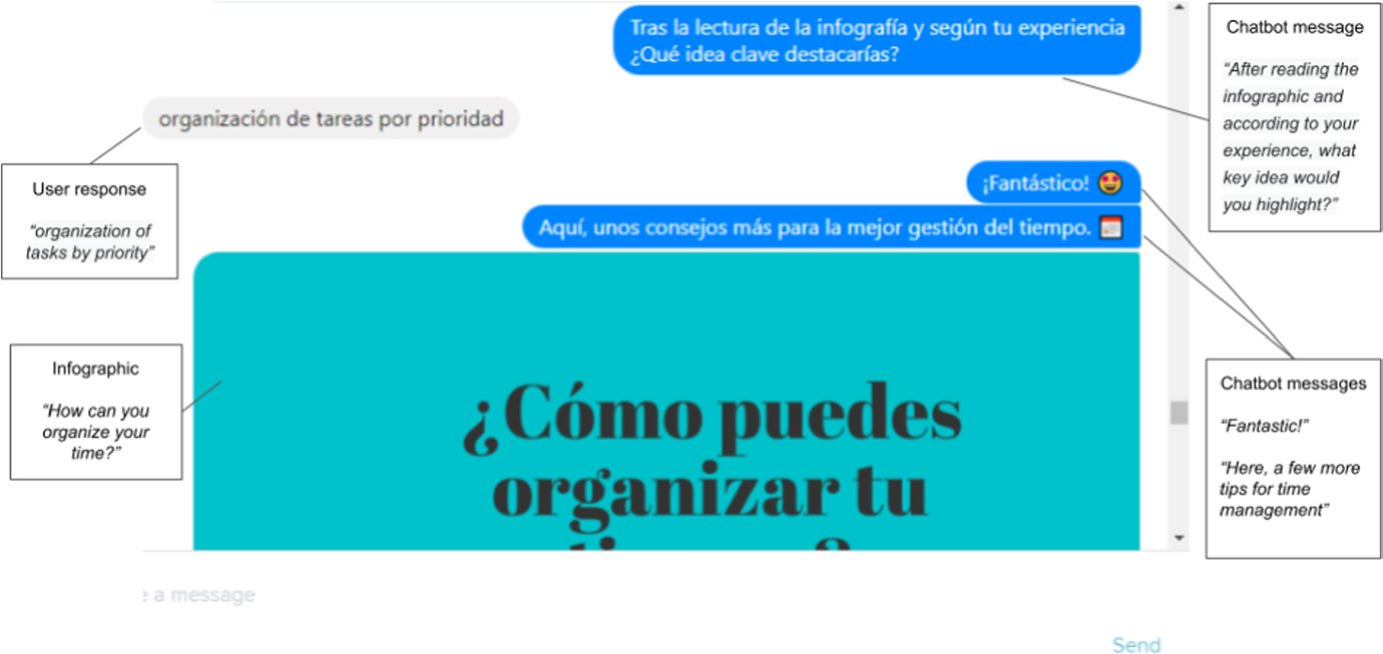
Infographic embedded in the EDUguia chatbot

The students would access the EDUguia chatbot through the university online campus and interact with it by answering questions. In order to support brief and smooth interactions, students would answer the chatbot questions by selecting from a range of predefined options. To support richer expression, in some cases it was also considered desirable to enable free text answers. The interface dialogue style aims to foster reflection on the part of the user and, at a certain point, suggests various tips in the form of infographic resources, as shown in Fig. 5. These resources were specially designed to accompany the development of learning self-regulation skills.

After the shaping of the chatbot into a functional prototype, lecturers were positive about the chatbot’s potential for supporting the students' learning process, improving their performance and their abilities to manage time, reflect and ask for support to manage their academic activity. In the lecturers’ view, the chatbot provided valuable resources, and the tool interface and language were friendly and easy to understand. When possible, the suggestions for improvement were incorporated before the pilot tests. In the cases of more complex requests, such as translating the tool into another language, they were listed as iterations to perform after the first round of pilot tests. Students’ early feedback after using the tool reflected their appreciation for the advice and the additional resources provided through the chatbot. Some of the students’ comments confirmed the need to schedule synchronous sessions to discuss their experiences using the chatbot for supporting self-regulation, and to revise the chatbot contents based on the students’ background and field of knowledge. While further evaluation of the chatbot will be performed after the first round of pilot tests in eight courses from diverse bachelor studies, we consider this feedback can be taken as a sign of the collaborative design worth for aligning the tool with the expected beneficiaries’ needs and values, as well as to open the design process.

## Discussion

This section discusses the challenges that hinder chatbot collaboration and the strategies used to overcome those challenges in relation to the design and technology research literature. Based on this, we indicate several implications for practice when co-designing learning environments mediated by technology.

Like other research studies, we distinguished different phases in the collaborative design process (Barberá et al., 2017; Cober et al., 2015). While the differentiation between three moments (*Understanding*, *Defining*, and *Shaping*) helped us to analyze and reflect on the challenges, it is worth noting that the boundaries between phases are blurry, and it is not possible to understand them as separate, linear tasks. As designers, we were responsible for leading a process that has been often described as messy (Akama, 2009; Blackler et al., 2018; Sanders & Stappers, 2008). Such messiness has an impact on collaboration since it makes communication and tracking decisions harder.

Traditionally, supporting mutual understanding between different stakeholders has been one of the key missions of participatory processes (Sanoff, 2011). Navigating stakeholders’ diverse and even opposing needs and creating consensus is not easy and requires participants to creatively explore their differences through shared discovery (Atlee, 2003; Sanoff, 2021). For this to happen, it is critical to find strategies that help reveal stakeholders’ tacit knowledge. As participatory design scholars have noted, some of these strategies might consist, for instance, of the conversation analysis of users’ dialogue (Luck, 2003) or asking participants to express their ideas by doing and enacting (Akach et al., 2021; Sanders et al., 2010; Spinuzzi, 2005). While these strategies have been widely adopted in participatory and co-design processes, it is worth noting that the level of analysis required for unveiling implicit meanings takes time and a certain distance to critically reflect on it. As designers and researchers involved in the EDUguia chatbot design, we were attentive to the dialogues that arose during the workshops, as well as their phrasings when providing textual feedback. However, undertaking such a thorough examination of the participants’ expressions was very challenging during the design process, and we only could do it when analyzing the session's documentation. Considering this circumstance to be quite common in research and innovation projects, we advocate the adoption of strategies that actively engage participants in making explicit the ideas and knowledge they take for granted. Developing shared vocabularies between designers and stakeholders can help to share meanings and create mutual understanding (Luck, 2003; Martin-Hammond et al., 2018). Based on our experience in technology-enhanced learning, we believe that the identification of specific strategies that contribute to support mutual understanding about learning and technology among education stakeholders would contribute to making learning technology design processes more open and democratic.

While mutual understanding is key in collaborative decision making, it does not impede conflicts from occurring. Participatory design scholars have devoted attention to conflicts and disagreements in design processes, outlining various mechanisms to guide decision-making in these situations (Bratteteig & Wagner, 2012; Hendriks et al., 2018; Pedersen, 2020). Among these mechanisms, building trust has been pointed out to support equitable collaborations and power sharing (Clarke et al., 2021; Hillgrenet al., 2011; Pirinen, 2016; Warwick, 2017). Interpersonal relations have been considered key for trust-building and there have been calls for supporting informal exchanges and spending time with stakeholders, beyond the project-focused activities. In the context of EDUguia, we found it challenging to build strong interpersonal relations with students, since we were not able to have the same level of regular interaction as with other stakeholders, like, for instance, the lecturers, throughout the design process. Our trust-building actions with students were limited to the online co-design workshops, in which informal conversations were harder than in face-to-face interactions. Given these circumstances, our efforts for building trust consisted in being open about the project goals and process, explicitly recognizing them as experts of their learning processes, showing appreciation for their insights and inviting them to give feedback at later stages of the project.

Because of time and resource constraints, we could not involve all stakeholders in all decisions, as we would have wished. Since this may be a common situation in design, we want to open a discussion on democratic decision-making in learning technology design since this has significant implications for practice. To this end, we consider it crucial to distinguish between different types of decisions and how to support trust in the decision-making process that leads to each of them. Following Bratteteig and Wagner, design decisions can be classified “big decisions and small decisions, decisions internal to the project and related to the outside world, and decisions that might be called non-decisions.” (Bratteteig & Wagner, 2012, p.41). In this paper, we have discussed the big and small decisions that were taken based on the requirements identified for the EDUguia chatbot design. We acknowledge that many more decisions were taken, but we think the ones presented are representative enough to highlight the complexities that arise in this type of process.

According to Bratteteig and Wagner, big design decisions relate to values and concepts which reflect in the visions guiding the project, as well as in the ways about how such visions are implemented (2012). In the design of EDUguia, the big decisions stemmed from the requirements identified through the co-design workshops were Use across disciplines, Theory-based, Immediate feedback, Adaptation to existing technology habits, Accessible and inclusive, Limited personal data collection and Data transparency. These decisions were made by consensus. In the cases in which we noticed different views among the stakeholders, we made them explicit to open a negotiation and better understand their priorities.

Small decisions in the EDUguia design were Ease of understanding and Brief and smooth interactions. As Bratteteig and Wagner note (2012), users are frequently involved in small decisions regarding formal aspects to ensure the final solution is familiar and easy to make sense of. Considering that the EDUguia chatbot was addressed to students, we prioritized their views for these decisions, which were made by asking them to vote between different options. After the voting, we opened a discussion about the reasons for selecting a particular option to ensure we were able to understand the rationale of their choices. Based on these criteria, we created a chatbot style guide that we use as reference during the writing of the chatbot script. The creation of an intermediate material such as the EDUguia style guide reflects the idea that design decisions are interrelated in diverse ways (Bratteteig & Wagner, 2012). From this perspective, the creation of intermediary objects can help to build trust in the design process by making explicit the rationale that guides further design decisions.

## Implications for practice and concluding remarks

In this paper we have presented diverse challenges faced in the collaborative design of the EDUguia chatbot, and we have discussed how they affected the decisions that led to the definition of the chatbot design requirements and the shaping of the tool. We consider the differentiation between big and small decisions relevant, because not all decisions have the same importance. When the stakeholders’ participation is limited, as designers we need to identify which are the important decisions in which it is crucial that stakeholders are involved. For this to occur, opening a discussion with stakeholders on which are the key aspects on which big decisions need to be taken, distinguishing them from less relevant issues is necessary. The differentiation between big and small decisions is also valuable to assess when we need to ensure a consensus is reached. From our perspective, big design decisions require consensus and thus the decision stream needs to stop until an agreement is reached. This might require developing strategies to support communication, mutual understanding, and collaboration. In contrast, for small decisions we consider that asking for feedback at certain points might be enough. As described in this paper, the use of intermediate objects can help open the design process documenting agreements and scaffolding decision-making.

While the study presented in this paper is limited in terms of the number of participants and the technologies examined, we believe the implications for practice outlined in this study are valuable for designers and researchers developing learning technologies such as chatbots for education contexts. With this piece of work, we seek to nurture further research and innovation work using collaborative design and democratic participation in learning technology design. In this regard, we advocate that researchers, designers and developers engage in a collective reflection on big and small decisions in collaborative design, and co-create strategies to overcome the obstacles that hinder collaboration and mutual understanding when designing learning technologies.

## Supplementary Information

Below is the link to the electronic supplementary material.

Supplementary file1 (PDF 403 kb)

## Data Availability

The datasets used and/or analyzed during the current study are available from the corresponding author on reasonable request.

## Footnotes

1 See Fernández-Ferrer et al. (2021) for more detailed information on the co-design workshops.
