# Supplementary material for: Designing learning technology collaboratively: Analysis of a chatbot co-design
Source: Educ Inf Technol (Dordr). 2022 Jun 24;28(1):109–34. doi: 10.1007/s10639-022-11162-w (PMC9226288; doi:10.1007/s10639-022-11162-w)
Supplement: Supplementary file 1 — Supplementary file1 (PDF 403 kb) [file 10639_2022_11162_MOESM1_ESM.pdf]

APPENDIX

Questionnaires to the students attending the chatbot co-design workshop 2
Rubric for competence level development of learning skills based on the srl phases of Zimmerman’s cyclical model (3rd version) 5
EDUguia chatbot style guide 8
Questionnaire to assess EDUguia chatbot prototype 11

QUESTIONNAIRES TO THE STUDENTS ATTENDING THE CHATBOT CO-DESIGN WORKSHOP

A. Before the workshop

A0. Degree

A1. Which device do you use MOST to access class content?
- Computer
- Mobile Phone
- Tablet
- Other

A2. Would you like to access the chatbot via the virtual campus itself?
- Yes
- No
- I do not know

A3. If you answered "no" or "I don't know" to the previous question, where would you like to access the chatbot?

A4. Have you ever been involved in a chatbot experience or service?
- Yes
- No
- I'm not sure

A5. If you answered "yes" to the previous question, which specific experiences or services do you remember in a chatbot environment?

B. After the workshop

B1. Is this your first time participating in a co-design workshop?
- Yes
- No
- I'm not sure

B2. If you answered "yes" to the previous question, could you contextualize your previous experience in a co-design workshop and tell us how it went?

B3. Why did you decide to participate in the co-design session and what were your expectations?

B4. Rate the following items and statements about the April 15 co-design session from 1 (not at all) to 5 (totally agree) 5:

Comfort:
- The atmosphere of the session made me feel comfortable and free to express my ideas at all times.

Participation:
- The kind of experience has allowed me to participate and intervene on the issues to be discussed.

Learning:
- During the session I was able to learn and acquire new concepts making the experience enriching for my personal and/or professional development.

Satisfaction:
- I feel satisfied with the approach and overall dynamics of the session.

B5. To what extent do you think it would be interesting for this type of collaborative design dynamic with students to be promoted also at the institutional level in the university (for example, for the design of training programs, for the decision on the strategic plan itself or of the institution, etc.)?
- A lot
- Quite a lot
- Fairly
- Little

B6. Would you like to participate in the decision on the tools used academically at your university?
- Yes
- No
- I do not know

Thank you very much for your collaboration with us!!!

Finally, we would like to ask you two questions about the future of your participation in our research project:

1. Would you like to know more about the chatbot development process later?
   If your answer is "yes", please let us know where you would like us to contact you.

2. If necessary, could we contact you again to work with us again when the project is at a later stage?
   If your answer is "yes", please let us know where you would like us to contact you.

RUBRIC FOR COMPETENCE LEVEL DEVELOPMENT OF LEARNING SKILLS BASED ON THE SRL PHASES OF ZIMMERMAN'S CYCLICAL MODEL (3RD VERSION)

	INDICATOR	Expert	Advanced	Intermediate	Starter
**FORETHOUGHT PHASE**	**TASK ANALYSIS**<br><br>Analyse the characteristics of the task or activity: what to do, what targets/results are expected and by which criteria their achievement will be assessed.	Formulate their own learning objectives, integrating them into those of task or activity. At the same time, strategic planning is established that takes account of the most appropriate procedures and resources, and all the characteristics of the task or activity, enabling it to run within the period set successfully and effectively.	Consider and prioritise the different objectives of the task or activity considering their difficulty, implementation time, assessment criteria and the level of achievement they want to achieve, presenting the first planning phase with the appropriate procedures and resources.	Have some specific difficulty but, in general, understand what needs to be done for the task or activity and identify the assessment criteria they will be evaluated and the level of achievement they want to attain by making a first proposal for objectives, but proper planning is lacking.	Have many difficulties understanding what to do with the task or activity and have difficulty identifying the goals and level of achievement they want to attain and are unaware of most assessment criteria, all of which affects planning.
	**SELF-MOTIVATION**<br><br>Analyse the value of the task or activity, and this determines the degree of motivation and effort involved and determines the steps to follow depending on the level of perfection to be achieved (or the results).	Based on the objectives set in relation to the performance of the task or activity, they can identify what results are expected and what capabilities must be achieved successfully. Therefore, expectations are high but they know how to be self-motivated and how to plan so as to effectively overcome the limitations.	Based on the objectives set in relation to the performance of the task or activity, they can identify most of the results expected and most of the capabilities to successfully achieve them, so expectations are high, although there is some difficulty knowing how to self-motivate and planning to do so effectively.	Based on the objectives set in relation to the performance of the task or activity, they can vaguely identify what results are expected and some of the capabilities to be able to successfully achieve them, so they have expectations, but it is hard to see how to motivate themselves to achieve them so effectively.	Based on the objectives set in relation to the performance of the task or activity, they have many difficulties in identifying what results are expected and what capabilities are required to be able to achieve them successfully, so expectations are low, and not much interest is shown in analysing their limitations and possibilities.

**PERFORMANCE PHASE**	**SELF-OBSERVATION**<br><br>Monitors what is being done and compares it to some expert model of execution or quality external reference.	Monitor and compare very closely the similarities and differences between their implementation and some expert model or quality external reference; justifies most of the strengths and weaknesses of this realization and reflect and broadly propose the most relevant adequacy and/or improvement of actions.	Monitor in detail and compare similarities and differences between their execution and some expert models or quality external references; justify strong points and weak points of their execution and identify highly relevant adequacy actions and/or improvements.	Monitor and compare generally what they are doing with some expert models or external quality references and detect some aspects to be improved.	Monitor and are able to describe what is being done, but do not make comparisons with any expert model or external reference of quality or indicate appropriate actions and/or well-founded improvements.
	**SELF-CONTROL**<br><br>Maintains the focus and interest in performing the task by using metacognitive and motivational strategies.	Use diverse resources and strategies for learning (requesting help, creating maps or mental images, study techniques, time and working environment management, etc.) that allow the learner to focus on the task and maintain a good level of concentration and interest throughout the execution process.	Use diverse resources and strategies for learning (requesting help, creating maps or mental images, study techniques, time and working environment management, etc.) to maintain concentration and interest during most of the execution process.	Use some resources and strategies for learning (requesting help, creating mental maps or images, study techniques, time and working environment management, etc.) that they incorporate in an inadequate way to the context and characteristics of the task, and this does not allow them to focus attention and interest properly at times in the execution process.	Use too few strategies for learning (requesting help, creating maps or mental images, study techniques, time and working environment management, etc.) and have difficulty focusing on tasks and maintaining concentration and interest.

**SELF-REFLECTION PHASE**	**SELF-JUDGEMENT**<br><br>Evaluates the execution of the task or activity that has been performed.	Reflect and value the effectiveness of the learning process based very clearly on what they have done and argued, based on the quality criteria and the level of expectations that they have set, have done so by identifying and mentioning in a critical and justified manner the causes of success or failure.	Reflect and make judgments about what they have done clearly and argumentatively, based on the quality criteria and the level of expectations that they have set, and identify some of the causes of success or failure.	Reflect in depth and evaluate what they have done based on the quality criteria and the level of expectations they have set for themselves, but with little argument and without clearly identifying what success or failure is.	Observe what they have done but are unable to have any thoughts or make assessment based on the quality criteria and the expected objectives/results, and therefore they make no mention of what success or failure is due.
	**SELF-REACTION**<br><br>Reacts emotionally and cognitively to the assessments made about the successes and the failures of the task or activity performed and the ability to transfer it to future learning.	Analyse the results of the task or activity performed from the point of view of satisfaction, assessing their successes and failures as opportunities to improve, clearly identifying the strategies they must implement to improve now and in future learning.	Analyse the results of the task or activity performed from the point of view of satisfaction, assessing their successes and failures as opportunities to improve, and identify some of the strategies that should be implemented.	Analyse the results of the task or activity performed from the point of view of satisfaction, assessing their successes and failures as opportunities to improve, but they do not identify the strategies that should be implemented.	Have difficulties to analyse the results of the task or activity carried out from the point of view of satisfaction, and to assess their successes and failures as opportunities to improve.

EDUGUIA CHATBOT STYLE GUIDE

0.Objectives

- Support the student to self-organize and manage their resources to carry out an academic task successfully.
- Provide recommendations and additional resources to make the student aware of the importance of self-regulation.

Avoid:

- That the chatbot is understood as an evaluation tool, with accessible information for the teacher.
- That the chatbot is understood as a tool aimed at solving specific doubts related to content, or practical information (such as: dates and delivery format...etc.)

1.Language

First option: Spanish

Pay special attention: non-sexist and inclusive use of language
Specific Resources for Higher Education:

- University of Barcelona criteria on non-sexist use of language: https://www.ub.edu/cub/criteri.php?id=2510

2. Dialogue

2.1 Introduction

- This message is included when the chatbot is opened for the first time.
- It is clearly identified that the interaction occurs with a bot.
- The function of the chatbot is specified, as well as the data that is collected for research purposes (non-academic).
- The student is informed of their right to withdraw from the investigation.

2.2 Welcome & Farewell

Welcome:

- Bot identification, indicating that it is an automated conversational interface.

Farewell:

- The student is the one who decides to end the conversation. Ending the chat is an option to choose (continue vs end) that appears once the student has consulted one microcontent.
- Before ending the session, the student is asked to rate the interaction.

2.3 Style
Friendly, polite.
Some expressiveness is included by:

- Signs of approval through emojis.
- Use of terms such as “correct, perfect, great…”
- Exclamations!

Positive and constructive tone through:

- Use of recommendations.
- Recognition of relevant knowledge/experience of students.

Empathic tone through:

- Inclusion of questions about the level of difficulty that a certain task represents for them, how they feel...

2.4 Texts
Short sentences and simple texts (1 sentence per speech bubble).
Predefined answers to select:

- The number of possible answers: between 2 and 4.
- The text of the answers is concise and clear.
- After selecting an answer, the chatbot includes some reference to the chosen option as confirmation.

Avoid:

- Long paragraphs.
- Sentences with several subordinates.
- Closed questions that do not contribute to advancing the conversation or provide any relevant information for the student.
- Academic jargon.

2.5 Additional resources (microcontents)
Each resource should be identified by a concise and self-explanatory title.
When the title is accompanied by more text, the “bold” style is used for the title.

Distinguish between different types of resources:

- Instructions, guidance on how to deal with a certain task.
- Specific information, although with more elaboration than the instructions.
- Tools (if tools are included, opt for free software. If it is not free software, please specify, as well as the business/payment model. Avoid recommending services whose servers are outside the EU).
- Questions for self-reflection.

Concept maps, diagrams, and schematics are included in image format.

Avoid:

- Inclusion of links to external resources.

3. Visual resources

3.1 Emojis

UTF8
Use emojis in moderation.
Use emojis to express:

- Approval
- Support
- Other expressions
- Key concepts

3.2 Images

Use of colour images.
Use of colours in infographics and charts to highlight key concepts.

3.3 Videos

The use of videos is ruled out for the time being. If it is considered essential for the micro-contents, specifications will be defined.

3.4 Audios

The use of videos is ruled out for the time being. If it is considered essential for the micro-contents, specifications will be defined.

QUESTIONNAIRE TO ASSESS EDUGUIA CHATBOT PROTOTYPE

A. About the conversational interface

A1. Rate from 1 (not at all) to 5 (totally agree) the following statements about the perceived usefulness of the chatbot:

- It can help students in their learning process
- It can help students to complete assignments on time
- It can improve student performance
- It can be useful for students to reflect on their learning process
- It can be useful for students to know when to ask for help
- It can be a distraction for students when performing other tasks
- It may involve excessive effort for students

A2. Based on your browsing experience, rate the chatbot from 1 (not at all) to 5 (totally agree):

- It is clear and understandable
- Dialogues are too long
- The chatbot language is friendly
- I found it awkward to use
- It provides useful information
- It's easy to use
- Dialogues are boring
- The answer options are adequate
- The content of the chatbot is academic
- I have not felt confident using the chatbot

A3. What do you think is the most useful feature of the chatbot?

A4. What do you think is the least useful feature of the chatbot?

A5. Do you have any concerns about the use of the chatbot? Which?

B. About the microcontents (infographics)

B1. Rate from 1 (not at all) to 5 (totally agree) the following statements in relation to the infographics of the chatbot - which are a self-made resource made ad hoc for the project -.

- They provide useful information
- They can help students in their learning process
- The resources offered are easy to implement in relation to the task of my subject
- They are useful as academic recommendations
- The text is too long
- Language is friendly
- Language is understandable
- Content is overly academic
- They invite you to view them
- The design (style, typography, colors, icons…) seems appropriate to me

B2. Do you have any suggestions for improving infographics? Which one?
If you have any contribution on any of the infographics in particular, you can refer to it by indicating the title of it.
